# Human platelet lysate stimulates neurotrophic properties of human adipose-derived stem cells better than Schwann cell-like cells

**DOI:** 10.1186/s13287-023-03407-3

**Published:** 2023-07-21

**Authors:** Stefania Brambilla, Martino Guiotto, Enrica Torretta, Ilaria Armenia, Matteo Moretti, Cecilia Gelfi, Silvia Palombella, Pietro G. di Summa

**Affiliations:** 1 Cell and Tissue Engineering Laboratory, IRCCS Istituto Ortopedico Galeazzi, Via C. Belgioioso 173, 20157 Milan, Italy; 2 Department of Plastic and Hand Surgery, Centre Hospitalier Universitaire Vaudois (CHUV), University of Lausanne (UNIL), Lausanne, Switzerland; 3 Laboratory of Proteomics and Lipidomics, IRCCS Istituto Ortopedico Galeazzi, Via C. Belgioioso 173, 20157 Milan, Italy; 4 Instituto de Nanociencia y Materiales de Aragón, CSIC-University of Zaragoza, C/ Pedro Cerbuna 12, 50009 Zaragoza, Spain; 5 Regenerative Medicine Technologies Laboratory, Laboratories for Translational Research (LRT), Ente Ospedaliero Cantonale (EOC), Via F. Chiesa 5, 6500 Bellinzona, Switzerland; 6 Service of Orthopaedics and Traumatology, Department of Surgery, EOC, Lugano, Switzerland; 7 Euler Institute, Faculty of Biomedical Sciences, USI, Lugano, Switzerland; 8 Department of Biomedical Sciences for Health, University of Milan, Milan, Italy

**Keywords:** Adipose-derived stem cells, Human platelet lysate, Schwann cells, Peripheral nerve injury, Orthopedic trauma

## Abstract

**Background:**

Trauma-associated peripheral nerve injury is a widespread clinical problem causing sensory and motor disabilities. Schwann cells (SCs) contribute to nerve regeneration, mainly by secreting nerve growth factor (NGF) and brain-derived neurotrophic factor. In the last years, adipose-derived stem cells (ASCs) differentiated into SCs (SC-ASCs) were considered as promising cell therapy. However, the cell trans-differentiation process has not been effectively showed and presents several drawbacks, thus an alternative approach for increasing ASCs neurotrophic properties is highly demanded. In the context of human cell-based therapies, Good Manufacturing Practice directions indicate that FBS should be substituted with a xenogeneic-free supplement, such as Human Platelet Lysate (HPL). Previously, we demonstrated that neurotrophic properties of HPL-cultured ASCs were superior compared to undifferentiated FBS-cultured ASCs. Therefore, as following step, here we compared the neurotrophic properties of differentiated SC-like ASCs and HPL-cultured ASCs.

**Methods:**

Both cell groups were investigated for gene expression level of neurotrophic factors, their receptors and neuronal markers. Moreover, the expression of nestin was quantitatively evaluated by flow cytometry. The commitment toward the SC phenotype was assessed with immunofluorescence pictures. Proteomics analysis was performed on both cells and their conditioned media to compare the differential protein profile. Finally, neurotrophic abilities of both groups were evaluated with a functional co-culture assay, assessing dorsal root ganglia survival and neurite outgrowth.

**Results:**

HPL-cultured ASCs demonstrated higher gene expression of NGF and lower expression of S100B. Moreover, nestin was present in almost all HPL-cultured ASCs and only in one quarter of SC-ASCs. Immunofluorescence confirmed that S100B was not present in HPL-cultured ASCs. Proteomics analysis validated the higher expression of nestin and the increase in cytoskeletal and ECM proteins involved in neural regeneration processes. The co-culture assay highlighted that neurite outgrowth was higher in the presence of HPL-ASCs or their conditioned medium compared to SC-ASCs.

**Conclusions:**

All together, our results show that HPL-ASCs were more neurotrophic than SC-ASCs. We highlighted that the HPL triggers an immature neuro-induction state of ASCs, while keeping their stem properties, paving the way for innovative therapies for nerve regeneration.

**Graphical Abstract:**

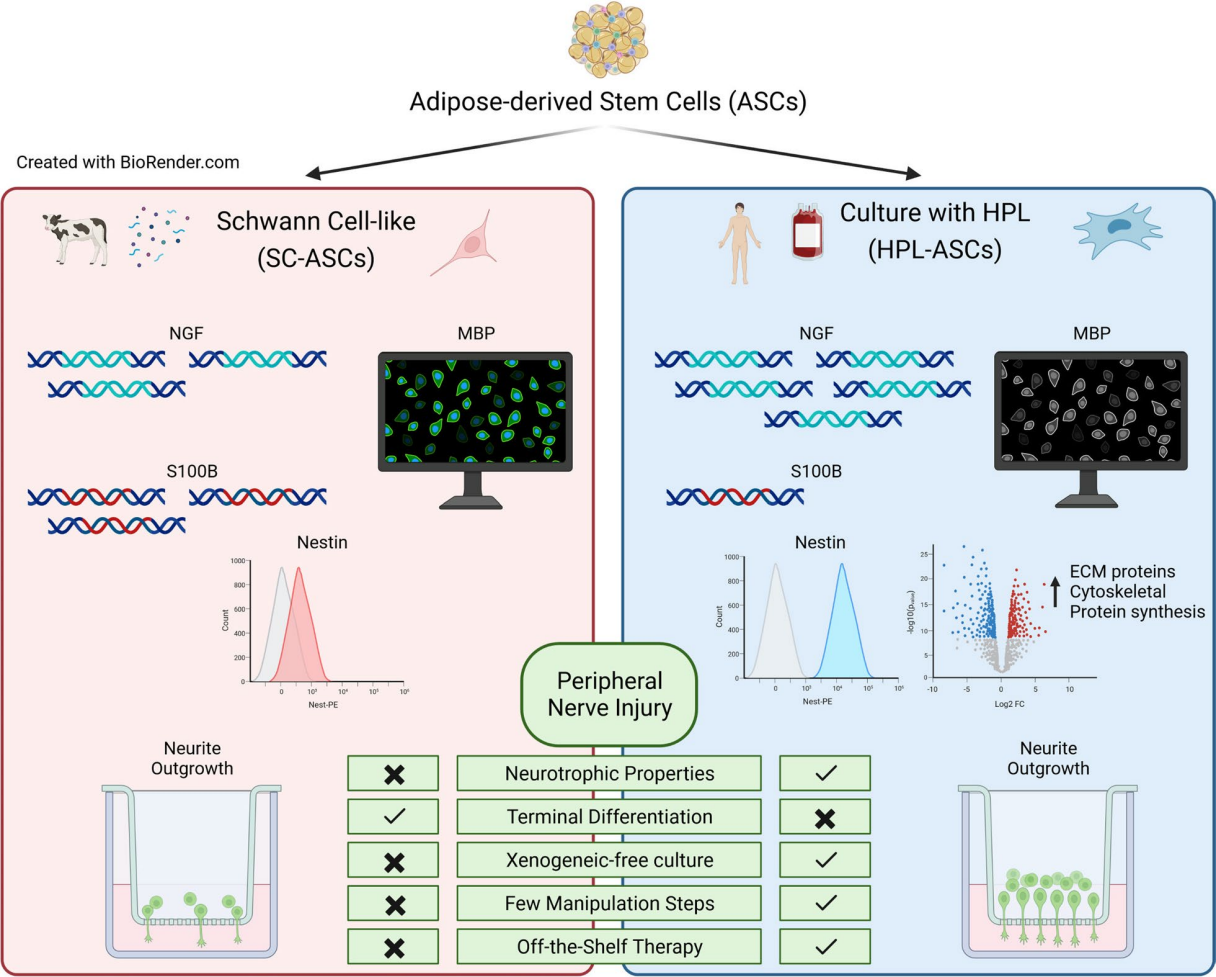

## Background

Trauma-associated peripheral nerve defects are a widespread clinical problem, and the incidence of sensory-motor disorders related to peripheral nerve injury (PNI) is quite high relevant all over the world [1, 2]. In fact, in Europe 300.000 cases of PNI occur annually, while in the USA approximately 360.000 people suffer from upper extremity paralytic syndrome annually. PNI is commonly related to sports, working activities or iatrogenic injuries to the upper and lower limbs themselves. PNI represents a major health problem that causes motor, sensory and autonomic disorders, often determing significant functional impairment and permanent disability [3]. The spontaneous intrinsic ability of the peripheral nervous system to repair and regenerate is limited, leading to low or absent functional mobility recovery without an appropriate treatment [4]. The current gold standard therapy in case of short gaps (< 2–3 mm) is an end-to-end anastomosis or an autologous nerve graft for longer gaps. However, these treatments often cause unsatisfactory clinical outcomes and poor recovery, mainly due to distal chronic denervation and fibrosis at the distal stump [5, 6].

Schwann cells (SCs) are one of the most representative cell types in the peripheral nervous system and their ability to revert to a repair competent state contributes significantly to the regeneration process after PNI [7]. There is emerging evidence of the therapeutic potential of SCs transplantation in promoting axonal repair and myelination in case of nerve defects [8]. Indeed, SCs are able to release neurotrophic factors, including nerve growth factor (NGF) and brain-derived neurotrophic factor (BDNF), stimulating the repairing process. Moreover, SCs offer a highly preferred substrate for axon migration and regrowth by expressing adhesion molecules and integrins on their surfaces that build up basement membranes for the support and guidance of the sprouting axons [9]. SCs also synthesize extracellular matrix (ECM) components, such as laminin and collagens, helping to promote axonal extension, supply the orientation and the adhesiveness for neurite outgrowth [10, 11]. All these advantages make SCs potential candidates for cell-based therapies as an alternative treatment for PNI. However, SCs have limited clinical applications, since their culture is time consuming and requires constant input of multiple growth factors. Besides that, these cells are not easily accessible without a nerve biopsy and the consequent sacrifice of a functional nerve [9].

Considering the SCs drawbacks in clinical translation, adipose-derived stem cells (ASCs) have been evaluated as a possible alternative for cell-based therapies for PNI. Indeed, ASCs are particularly attractive since they are abundant, easy to isolate, and can be obtained from waste materials from plastic surgeries. Moreover, ASCs have a high proliferation rate, great differentiation potential, immunomodulatory and angiogenic properties [12]. Furthermore, ASCs lack the major MHC class II molecules and express only low levels of MHC class I, allowing them to evade the host immune recognition [13]. Remarkably, ASCs are capable to create a favorable environment for nerve regeneration by producing a wide variety of neurotrophic factors which can enhance neurite outgrowth, provide neuroprotection, and support axonal regeneration both in vitro and in vivo [14, 15]. Several attempts were made to improve the ASCs neurotrophic function by differentiating them into SC-like cells (SC-ASCs). However, this topic is still inconclusive since the cell response is not fully elucidated with unclear benefits [16–21]. Additionally, the therapeutic use of SC-ASCs is hampered by the time-consuming differentiation protocols, limited clinical translation and concerns about their long-term stability [18, 22]. Thus, new cell culture methods need to be carried out to find new ways to improve ASCs neurotrophic properties.

The conversion of research-based protocols into GMP-compliant procedures requires protocols that have had careful consideration of all the risks and benefits for the patient and user [23–25]. In this context, animal sera commonly used for in vitro cell culture, such as FBS, are ill defined and pose a risk factor as source of xenogeneic antigens and possible transmitters of zoonotic infections, increasing the risk of immune reactions in the receiving patients and therefore, making animal-derived sera undesirable medium supplements for products intended for human use [26]. Moreover, FBS is subjected to batch-to-batch variability, thus affecting data reproducibility. In addition, ethical concerns have been raised on the current ways of collecting FBS that may cause suffering to the animals [27, 28]. Therefore, replacing animal-derived products with xeno-free alternatives would significantly enhance the safety and quality of ASCs for therapeutic approaches [29].

Recently, human platelet lysate (HPL) has been proposed as an alternative to FBS for in vitro culture of ASCs [24, 25]. HPL presents lower safety problems for recipient patients, since it is a xeno-free growth medium supplement of human origin and has very limited ethical issues compared to FBS production [30]. Moreover, HPL can be easily obtained from apheresis products or expired buffy coats as a pooled material, thus decreasing batch-to-batch variations and increasing data reproducibility [31]. Compared to FBS, HPL also contains a higher concentration of growth factors, such as platelet-derived growth factor (PDGF), basic fibroblast growth factor (bFGF), and transforming growth factor-α (TGF-α) allowing faster cell expansion, reducing in turn time and costs [32–34]. Recent evidences strongly support the use of HPL to culture ASCs, since not only it maintains the expression of stem cell markers and the differentiation potential, but also sustains cell viability, enhances proliferation, delays senescence, ensures cell genomic stability, and immunomodulatory properties [24, 35, 36].

We have already demonstrated that compared to FBS, HPL could increase the neurotrophic properties of ASCs, in terms of secretion of BDNF and stimulation of dorsal root ganglia (DRG) elongations [14]. Together with this, another recently published paper described the enhanced neurotrophic potency of ASCs in response to HPL in promoting axonal outgrowth to levels comparable to rat SCs [16]. All together, these data suggest that neuroregenerative potential of ASCs could be increased by the presence of HPL compared to classical FBS-cultured cells. Therefore, we hypothesized that HPL-cultured ASCs could have the same neurotrophic potential of SC-ASCs, with the advantages of using a GMP-ready protocol and without the drawbacks of the differentiation steps. Firstly, we evaluated the expression of typical SC markers, including nestin and S100B, to test the neural commitment of ASCs. Subsequently, we performed a functional co-culture with DRG to assess the neurotrophic potential of HPL-cultured ASCs compared to SC-ASCs. With the demonstration of our hypothesis, we would contribute to find a new cell-based therapy for the treatment of trauma-induced PNI.

## Methods

### Experimental design

Before isolating ASCs, each adipose tissue sample was divided into two parts and manipulated separately with FBS or HPL, respectively, in order to remove any possible contaminant effect. After initial expansion, ASCs cultured in the presence of FBS were differentiated into SC-like (SC-ASCs) and compared to HPL-cultured ASCs (HPL-ASCs). ASCs obtained from both cultures were used to evaluate the expression of neural markers and subsequently in the functional assay with DRG. All the experiments were performed with ASCs at low passage (P3) in order to reduce the selections of ASCs subpopulation. No antibiotics or antifungal were used in any culture media, as per GMP directions regarding the use of cell products for human use.

### Adipose stem cell isolation and culture

ASCs were isolated from subcutaneous adipose tissue obtained as waste material of abdominoplasty surgery of three healthy women (average age 40 ± 2). Cell isolation was performed as previously described [14]. Briefly, the adipose tissue was initially washed twice with PBS (Gibco) supplemented with 5000 U/mL penicillin–streptomycin (Gibco) to remove the excess blood and rinse the tissue from any contaminant debris. Subsequently, the tissue was mechanically minced and enzymatically digested with 0.15% (w/v) animal-free collagenase A (2 mL/g, Worthington Biochemical Corporation) at 37 °C for 1 h in agitation. The sample was split in two parts, and the collagenase action was inactivated with one volume of DMEM high glucose (Gibco) supplemented, respectively, with 10% FBS (Hyclone) or 5% HPL (Stemulate, Sexton Biotechnologies). The resulting solution was filtered through a 100 µm filter to remove undigested tissue. After three washing steps with PBS, the cell pellet was suspended in the corresponding complete medium added with 2 mM l-Glutamine (Gibco) and cultured at 37 °C and 5% CO_2_. The medium was changed after 24 h to remove unattached cells and then every 3–4 days until cell confluence. Cell morphology in the two conditions was checked daily under phase-contrast microscope (CKX41 Inverse Microscope Olympus, Hamburg, Germany).

### Treatment of FBS-cultured ASCs with neurogenic factors

In order to obtain SC-ASCs, FBS-cultured ASCs were cultured in the presence of neurogenic factors as previously described [20, 22]. In particular, ASCs were cultured in complete medium with 1 mM β-mercaptoethanol (SigmaAldrich) for 24 h and with 35 ng/mL all-trans-retinoic acid (Peprotech) for the subsequent 72 h. Afterward, the medium was supplemented with 5 ng/mL PDFG (Peprotech), 10 ng/mL bFGF (Peprotech), 14 µM forskolin (Biogems), and 252 ng/mL GGF-2 (Peprotech) and changed every 72 h for an overall period of 14 days.

### RNA isolation and retrotranscription

In order to assess the expression of SC markers, the total RNA was isolated with TRIzol Reagent (Invitrogen) following manufacturer instructions. Briefly, about 1 × 10^6^ SC-ASCs and HPL-ASCs were suspended in 1 mL TRIzol Reagent and incubated 5 min at room temperature. After adding 200 µL chloroform, the samples were centrifuged for 15 min at 12,900 g at 4 °C. The resulting aqueous phase was transferred, and RNA was precipitated with 500 µL isopropanol. After incubation of 10 min at room temperature and centrifugation of 10 min at 12,900 g at 4 °C, the RNA pellet was washed once with 1 mL 75% ethanol. Subsequently, the RNA pellet was suspended in 20–50 μL nuclease-free water depending on size. RNA concentration was determined using NanoDrop N-100 (Thermo Fisher Scientific).

The first-strand cDNA was synthesized using the iScript cDNA Synthesis kit (BioRad) according to the manufacturer instructions. One microgram of total RNA was used as the initial template in a final reaction volume of 20 μL and subsequently diluted to the final concentration of 5 ng/μL. The thermocycler was set up as follows: 25 °C for 5 min, 42 °C for 30 min, 45 °C for 15 min, 85 °C for 5 min.

### Quantitative PCR

The gene expression analysis was performed with StepOnePlus Real-Time PCR system (Applied Biosystems). The reaction was set up using 10 ng cDNA, primers at final concentration of 300 nM each (primer sequences are reported in Table 1), and iTaq Universal SYBR Green Supermix (Biorad) in a final volume of 20 μL. The reactions were carried out in triplicates. The thermocycler program had an initial hot start step at 95 °C for 20 s, followed by 40 cycles at 95 °C for 3 s and 60 °C for 30 s. To confirm primer specificity, a melting curve analysis was performed after each amplification. Reference genes were selected as described previously [37]. The level of target genes was expressed as 2^−ΔΔCt^ compared to the control sample SC-ASCs, arbitrarily set at 1 and statistically analyzed with t Student’s test.

**Table 1:** Sequences of primers used to evaluate SC markers

Reference gene	Primer sequence 5′ → 3′	Target gene	Primer sequence 5′ → 3′
*RPL13A*	Fw: TATGAGTGAAAGGGAGCC Rv: ATGACCAGGTGGAAAGTC	*NGF*	Fw: TGAAGCTGCAGACACTCAGG Rv: AGAATTCGCCCCTGTGGAAG
*RPS18*	Fw: GAGGTGGAACGTGTGATC Rv: GGACCTGGCTGTATTTTC	*BDNF*	Fw: AACATGTCCATGAGGGTCCG Rv: CAGTCTTTTTGTCTGCCGCC
*PPIA*	Fw: AACCACCAGATCATTCCTT Rv: GCGAGAGCACAAAGATTC	*GDNF*	Fw: TTTAGGTACTGCAGCGGCTC Rv: GCCTGCCCTACTTTGTCACT
*UBC*	Fw: CACTGGCAAGACCATCACC Rv: TCAACCTCTGCTGGTCAGG	*NTRK1*	Fw: GCCACATCATCGAGAACCCA Rv: CTCCCACTTGAGCACGATGT
*YWHAZ*	Fw: AGTCATACAAAGACAGCACGC Rv: TTCAGCTTCGTCTCCTTGGG	*NTRK2*	Fw: TCTGCTCACTTCATGGGCTG Rv: GTGGTGTCCCCGATGTCATT
*TBP*	Fw: GCCCGAAACGCCGAATATAA Rv: AAATCAGTGCCGTGGTTCGT	*NGFR*	Fw: CTGAGGCACCTCCAGAACAA Rv: ACAGGGATGAGGTTGTCGGT
*GUSB*	Fw: CGGCCTGTGACCTTTGTGA Rv: AGATCACATCCACATACGGAGC	*GFRA1*	Fw: CCACTCATGTTTTGCCACCG Rv: ACAGAGGTGTGTATTGCCCG
		*NEST*	Fw: GACCCTGAAGGGCAATCACA Rv: GGCCACATCATCTTCCACCA
		*S100B*	Fw: GGAAATCAAAGAGCAGGAGGT Rv: ATTAGCTACAACACGGCTGGA

### Quantification of nestin by flow cytometry

To quantify the level of the marker nestin, SC-ASCs and HPL-ASCs were stained with the specific antibody and evaluated by flow cytometry. In particular, both cell groups were treated with eBioscience™ Intracellular Fixation & Permeabilization Buffer Set (ThermoFisher Scientific) following manufacturer instructions. After being fixed and permeabilized, 500,000 cells were suspended in 100 µL of antibody solution consisting of the antibody anti-nestin (1:20, BioLegend, PE-labeled) diluted in MACS buffer (Miltenyi Biotec) and incubated for 10 min at 4 °C in the dark. The cell surface phenotype was subsequently assessed by CytoFLEX flow cytometer (Beckman Coulter), recording at least 100,000 events in the selected gate. For both groups, unstained cells were used as reference for autofluorescence. We recorded the parameters regarding cell dimension (FSC-A) and median fluorescence intensity (MFI) for both unstained and stained samples. The ratio of ΔMFI was calculated by dividing HPL-ASCs (stained MFI—unstained MFI) for SC-ASCs (stained MFI—unstained MFI) and used as indicator for antigen amount. Statistical analysis was performed with t Student’s test between the control group SC-ASCs and HPL-ASCs.

### Immunofluorescence staining of the neurogenic markers

About 10,000 cells/cm^2^ of both SC-ASCs and HPL-ASCs were cultured in µ-Slide 8 Well (Ibidi) for 24 h before fixation with 4% paraformaldehyde at room temperature for 10 min. Samples were blocked and permeabilized with 1% BSA (Sigma-Aldrich) and 0.2% Triton-X100 (AppliChem) in PBS for 20 min. Cells were incubated with the following primary antibodies: anti-MBP (1:500, Abcam, mouse monoclonal) and anti-s100β (1:100, Abcam, rabbit monoclonal) at RT for 2 h. Subsequently, cells were incubated for 1 h at RT with FITC-conjugated secondary antibody (anti-mouse or anti-rabbit, 1:200, Abcam, polyclonal). Cell nuclei were labeled with Hoechst 33,342 (Thermo Fischer) added to the secondary antibody solution at a final concentration of 8 μM. Samples were examined under fluorescence microscope at 10 × magnification (Olympus IX81).

### Proteomic analysis

Proteomic analysis was performed on both SC-ASC and HPL-ASC cells and their conditioned media, collected after a starvation period of 24 h. The cells were detached using Cell Dissociation Buffer enzyme-free (Gibco), incubated for 5 min at 37 °C and 5% CO_2_ and mechanically collected with a scraper. The media were collected and centrifuged 10 min at 15,500 g to remove cellular debris. The collected samples were stored at − 80 °C until processing. Cell pellets were resuspended in 2% SDS in 0.1 M Tris/HCl pH 7.6, 1 mM phenylmethanesulfonyl fluoride (PMSF), sonicated on ice and incubated at 95 °C for 3 min. After clarification by centrifugation at 16,000 g for 5 min at 20 °C, protein quantitation was determined by Pierce bicinchoninic acid (BCA) protein assay (Thermo Fisher Scientific). Supernatants were first mixed with cOmplete™ Protease Inhibitor Cocktail (Roche), concentrated with Pierce protein concentrators 30 K (Thermo Scientific) and mixed with 2% SDS in 0.1 M Tris/HCl pH 7.6.

Following the FASP (Filter-Aided Sample Preparation) protocol [38], 200 μg of proteins from each sample were placed in 30 kDa filters (Sigma-Aldrich) for in-tube reduction (100 mM DTT in 50 mM ammonium bicarbonate), alkylation (0.05 M IAA iodoacetamide in 8 M urea in 0.1 M Tris/HCl pH 8.5) and digestion with 1:50 (w/w) Trypsin Gold, MS grade (Promega, Madison, WI, USA) for 16 h at 37 °C.

The obtained peptides were subjected to LC–ESI–MS/MS shotgun analysis performed on a Dionex UltiMate 3000 HPLC System with an Easy Spray PepMap RSLC C18 column (150 mm, internal diameter of 75 μm; Thermo Fisher Scientific) with the following gradient: 5% acetonitrile (ACN) in 0.1% formic acid for 10 min, 5–35% ACN in 0.1% formic acid for 79 min, 35–60% ACN in 0.1% formic for 40 min, 60–100% ACN for 1 min, 100% ACN for 10 min at a flow rate of 0.3 μL/min. The eluate was electrosprayed into an Orbitrap Tribrid Fusion (Thermo Fisher Scientific) through a nanoelectrospray ion source (Thermo Fisher Scientific). The LTQ-Orbitrap was operated in positive mode in data-dependent acquisition mode to automatically alternate between a full scan (350–2000 m/z) in the Orbitrap (at resolution 60,000, AGC target 1,000,000) and subsequent CID MS/MS in the linear ion trap of the 20 most intense peaks from full scan (normalized collision energy of 35%, 10 ms activation). Isolation window: 3 Da, unassigned charge states: rejected, charge state 1: rejected, charge states 2+ , 3+ , 4+ : not rejected; dynamic exclusion enabled (60 s, exclusion list size: 200). Mass spectra were analyzed using MaxQuant software [38] (version 1.6.17.0). The initial maximum allowed mass deviation was set to 6 ppm for monoisotopic precursor ions and 0.5 Da for MS/MS peaks. Enzyme specificity was set to trypsin/P, and a maximum of two missed cleavages was allowed. Carbamidomethylation was set as a fixed modification, while N-terminal acetylation and methionine oxidation were set as variable modifications. The spectra were searched by the Andromeda search engine against the Homo Sapiens Uniprot sequence database (release 10.02.2021) [39]. Protein identification required at least one unique or razor peptide per protein group. Quantification in MaxQuant was performed using the built-in XIC-based label-free quantification (LFQ) algorithm using fast LFQ [40]. The required false positive rate (FDR) was set to 1% at the peptide, 1% at the protein and 1% at the site-modification level, and the minimum required peptide length was set to 7 amino acids. To “clean-up” secretome results, affected by contaminants released from dying or broken cells, proteins were filtered for showing at least 80% valid LFQ intensity values and considered as secreted when showing a fold change (mean LFQ intensity conditioned medium)/(mean LFQ intensity cell lysate) > 1.5 or which exclusively show LFQ intensities in the conditioned medium.

### Functional co-cultures with DRG

To verify the neural regenerative potential of SC-ASCs and HPL-ASCs, we set up a functional co-culture with rat DRG neurons. DRG were purchased cryo-preserved from Innoprot and cultured with the specific Neuronal Medium added with 1% neuronal growth supplement, 1% penicillin/streptomycin (Innoprot). The day of the experiment, SC-ASCs and HPL-ASCs were seeded at density of 20,000 cell/cm^2^ in non-treated 24 well plates pre-coated with 2 µg/mL poly-L-lysine (Sigma-Aldrich) 24 h before seeding DRG. The day after, DRG were thawed, counted and directly seeded at density of 5000/cm^2^ (ratio 1:4 with the corresponding ASCs group) and co-cultured for the subsequent 72 h. In order to consider all the possible confounding factors we established the following conditions:

- *Condition 1 (control 100%)* DRG alone with 100% Neuronal Medium;
- *Condition 2 (control 50%)* DRG alone with 50% Neuronal Medium and 50% FBS medium or HPL medium;
- *Condition 3 (direct co-culture)* DRG directly in contact with SC-ASCs or HPL-ASCs with 50% Neuronal Medium and 50% FBS medium or HPL medium, respectively;
- *Condition 4 (indirect co-culture)* DRG indirectly cultured with SC-ASCs or HPL-ASCs with 50% Neuronal Medium and 50% FBS medium or HPL medium, respectively, in transwell plates (pore dimension: 0.4 µm, Corning);
- *Condition 5 (conditioned medium)* DRG alone with 50% Neuronal Medium and 50% conditioned medium from SC-ASCs or HPL-ASCs, respectively.

### Immunofluorescence detection and analysis of DRG elongations

After 72 h of co-culture, DRG and ASCs were fixed with 4% paraformaldehyde for 10 min at room temperature. Subsequently, samples were blocked with 1% BSA (Sigma-Aldrich) in PBS and permeabilized with 0.2% Triton X-100 (Sigma-Aldrich) for 20 min at room temperature. Cells were stained with anti-βIII-tubulin (1:500, Abcam, rabbit monoclonal) and for 1.5 h at room temperature and subsequently with anti-rabbit Alexa Fluor 488-conjugated secondary antibody (1:1000, Thermo Fisher) for 1 h at room temperature in the dark. Cell nuclei were labeled with DAPI (Thermo Fisher) added to the secondary antibody solution at final concentration of 300 nM. Samples were examined at a 10× magnification under fluorescence microscope (Olympus IX81). For each DRG present at the end of the culture, one picture enclosing all the elongations was taken. Firstly, the number of surviving DRG in each condition was calculated. Subsequently, the total neurite length for each DRG was calculated with ImageJ software.

## Results

### HPL influences the expression of neuronal marker genes

We firstly evaluated the expression of neuronal marker genes in both SC-ASCs and HPL-ASCs. In particular, we analyzed the genes coding for factors typically secreted from SCs, that is NGF, BDNF and Glial Cell Derived Neurotrophic Factor (GDNF) and their receptors NTRK1 (high-affinity receptor for NGF), NTRK2 (high-affinity receptor for BDNF), NGFR (low-affinity receptor for NGF and BDNF), GFRA1 (receptor for GDNF). Moreover, we investigated the expression of NES (neuronal precursor cell marker, expressed primarily in neuronal cells), S100B (mature SC marker, involved in neurite extension), and GAP43 (nerve regeneration marker, highly expressed during neuronal development and axonal regeneration).

**Fig. 1:**
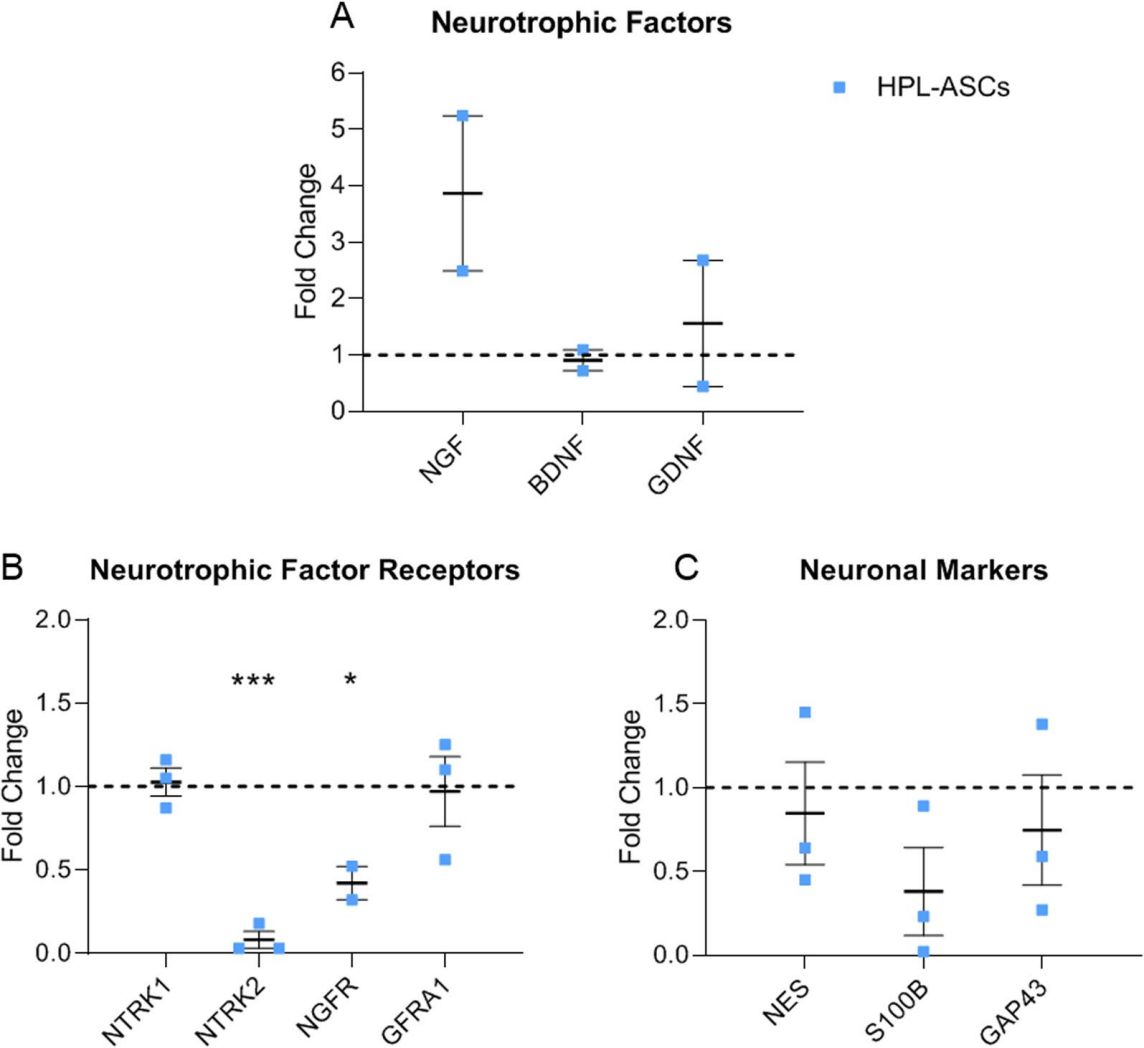
Gene expression of neuronal markers. **A** Genes coding for factors typically secreted from SCs (NGF, BDNF, and GDNF); **B** their receptors (NTRK1, NTRK2, NGFR, GFRA1); **C** and neuronal markers NES, S100B, and GAP43 were investigated. Results are expressed as fold change compared to control group SC-ASCs, indicated by the dotted line. HPL-ASCs expressed NGF more than SC-ASCs. Accordingly, the expression of NTRK2 and NGFR was statistically reduced in HPL-ASCs, as answer to the negative feedback induced by NGF. Moreover, S100B expression was decreased in HPL-ASCs compared to the control group. (*p < 0.05; ***p < 0.001)

We noticed that HPL-ASCs showed an increasing trend in the expression of NGF compared to SC-ASCs. This result could predict that HPL-ASCs are more neurotrophic than SC-ASCs. On the contrary, BDNF and GDNF were not differentially expressed between the two groups (Fig. 1A). Similarly, the expression of NTRK1 and GFRA1 was not altered by the presence of HPL. On the other hand, the expression of NTRK2 and NGFR was statistically decreased in the HPL-ASCs compared to SC-ASCs, indicating a negative feedback, induced by the high expression of NGF (Fig. 1B). Regarding the expression of NES and GAP43, no statistical difference was assessed between the two groups. On the contrary, we identified a not significant reduced expression of the gene S100B in HPL-ASCs compared to SC-ASCs, suggesting that HPL-ASCs were not terminally differentiated (Fig. 1C). These results support that HPL-ASCs were closer to immature SCs phenotype, thus indicating that HPL could induce a neuronal precursor state of ASCs while stimulating their neurotrophic properties.

### Nestin is more expressed in HPL-ASCs than in SC-ASCs

**Fig. 2:**
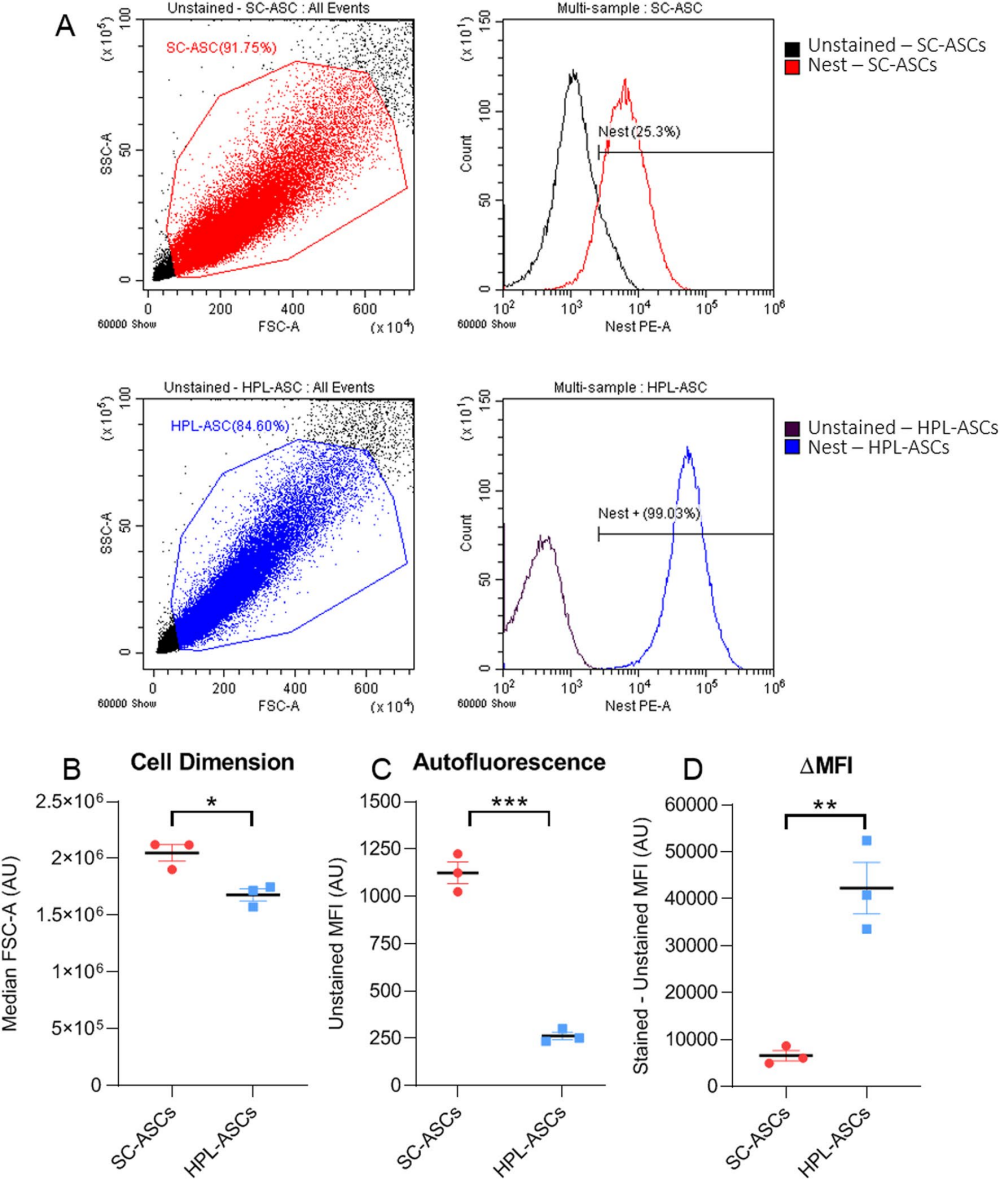
Quantitative levels of nestin by flow cytometry. **A** Nestin protein level was evaluated both in SC-ASCs and HPL-ASCs and compared to the corresponding unstained sample. Nestin was expressed in almost all HPL-ASCs and only in one quarter of SC-ASCs. **B** Parameters regarding cell dimension (FSC-A) indicated that HPL-ASCs were smaller than SC-ASCs. **C** The MFI of unstained samples indicated that autofluorescence of HPL-ASCs was lower than SC-ASCs. (**D**) The calculation of the ΔMFI suggested that nestin was expressed almost 7 times more in HPL-ASCs than in SC-ASCs. (*p < 0.05; **p < 0.01; ***p < 0.0001)

To study the neuronal commitment of SC-ASCs and HPL-ASCs, we quantitatively analyzed the expression of the marker nestin by flow cytometry. Surprisingly, only 25.3% of SC-ASCs expressed the protein Nestin, whereas almost all HPL-ASCs expressed this protein (99.03% positive cells) (Fig. 2A).

On the basis of the parameters analyzed, we can assert that HPL-ASCs were statistically smaller compared to SC-ASCs, as indicated by the FSC-A value which is 17.64% lower in HPL-ASCs than the control group (Fig. 2B). Correspondingly, the evaluation of the MFI of unstained samples confirmed that the autofluorescence of HPL-ASCs was 76.66% lower than SC-ASCs (Fig. 2C). Moreover, the ratio of the ΔMFI between the HPL-ASCs and SC-ASCs indicated that nestin was expressed almost 7 times more in HPL-ASCs than in SC-ASCs, supporting our hypothesis that HPL could sustain the neurotrophic properties of ASCs (Fig. 2D).

### HPL-ASCs are neuro-committed toward SCs phenotype

Since HPL contains a high amount of different growth factors involved in neuronal commitment and differentiation, we investigated whether ASCs cultured with HPL were neuronal committed or terminally differentiated as SC-ASCs. Specifically, we analyzed with immunostaining techniques two neuronal markers: Myelin Basic Protein (MBP), specific of myelinating processes, and S100B, a mature SC marker.

**Fig. 3:**
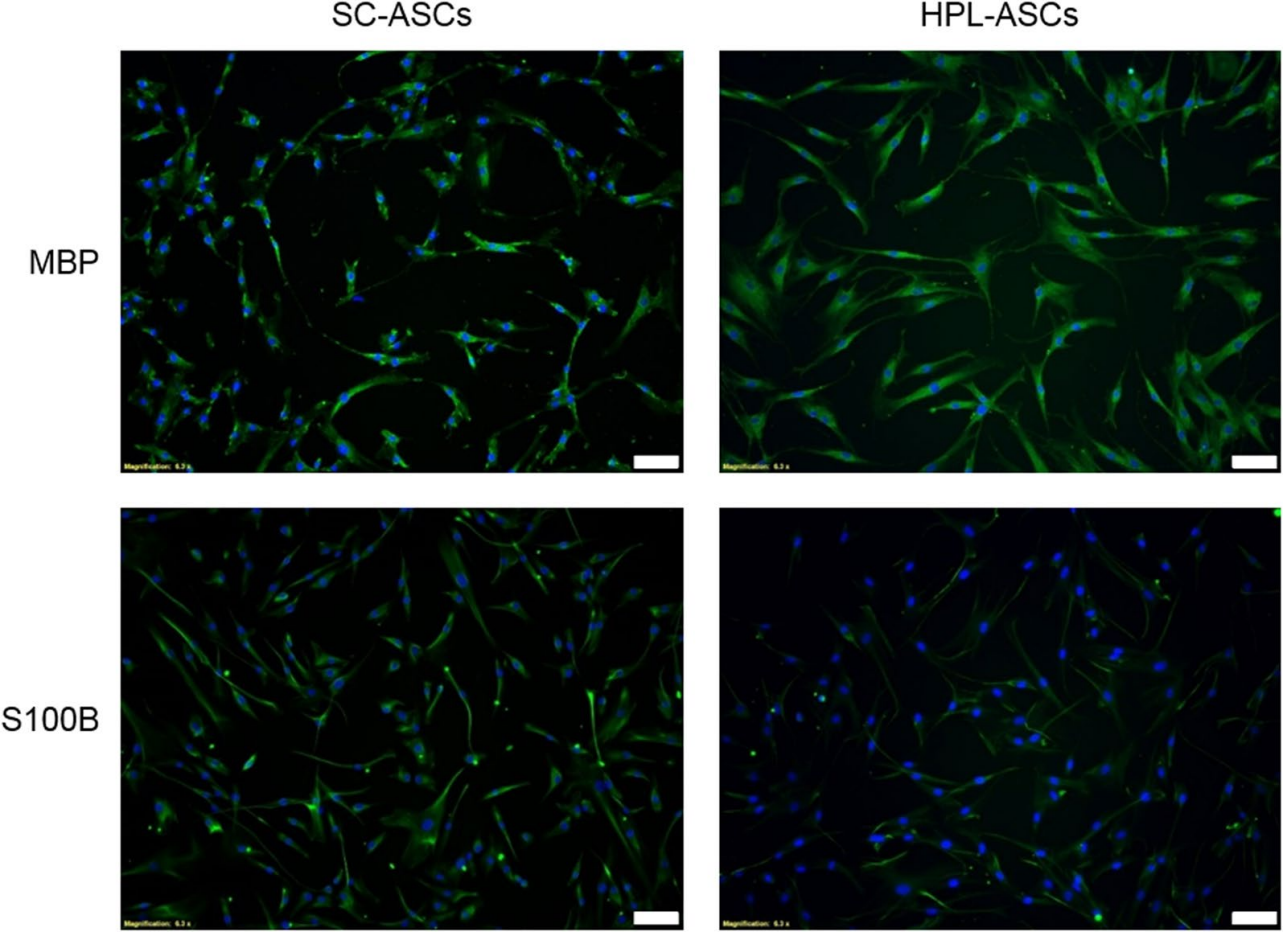
Immunofluorescence of MBP and S100B. Both SC-ASCs and HPL-ASCs were investigated for the presence of MBP (expressed during myelinating process) and S100B (mature SC marker). The SC-ASCs expressed MBP with a dotted membrane signal and resulted positive for S100B, indicating a successful and terminal differentiation. On the contrary, HPL-ASCs expressed MBP with a cytosolic signal and were negative for S100B, suggesting that these cells were only neuro-committed (white bars: 100 μm)

MBP was detected in SC-ASCs with a dotted signal compatible with membrane proteins as in active myelinating SCs. On the other hand, in HPL-ASCs MBP appeared to be more distributed in the cytosol typical of immature proteins. Moreover, the S100B SCs marker was detected in SC-ASCs as expected, indicating a successful and terminal differentiation of these cells. On the contrary, HPL-ASCs showed no positive signal for this marker (Fig. 3). Taking into account the previous results, this outcome suggested that HPL-ASCs were only neuro committed and not terminally differentiated into SCs, while possessing neurotrophic properties.

### ASCs cultured with HPL expressed more neurogenesis-involved proteins

Protein extracts from SC-ASCs and HPL-ASCs and from their conditioned media were analyzed by liquid chromatography coupled to electrospray tandem mass spectrometry (LC–ESI–MS/MS) and quantified by label-free approach. Student *t*-test revealed 35 changed proteins among the 1039 proteins identified in cells and 327 changed proteins among the 1760 proteins identified in the secretome. Among these, 255 proteins were considered as bona fide secreted due to the comparison with the cellular proteome.

**Fig. 4:**
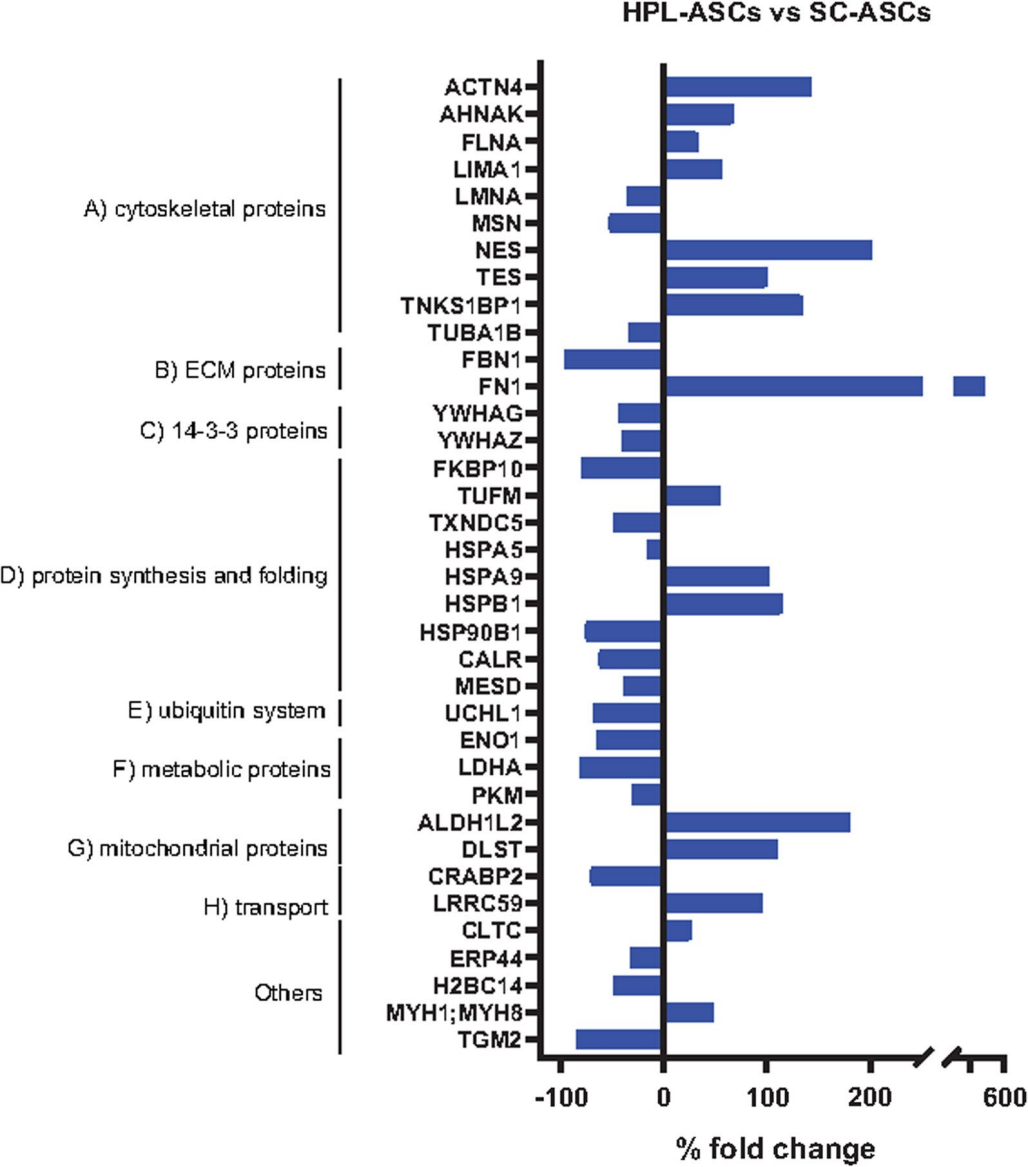
Proteomic analysis of HPL-ASCs versus SC-ASCs. Bars of differentially expressed proteins after label-free quantification (LFQ intensity % variation, *p*-value < 0.05, *q*-value < 0.05)

Results were displayed as differentially expressed in HPL-ASCs compared to SC-ASCs. Nestin was confirmed to be more expressed with a fold change of 200%. Other cytoskeletal proteins increased, including alpha-actinin 4 (ACTN4), neuroblast differentiation-associated protein AHNAK (AHNAK), filamin-A (FLNA), LIM domain and actin-binding protein 1 (LIMA1), testin (TES), and 182 kDa tankyrase-1-binding protein (TNKS1BP1), whereas prelamin A/C (LMNA), moesin (MSN), and tubulin alpha 1 chain (TUBAB1) decreased (Fig. 4A). Among ECM proteins, fibronectin-1 (FN1), involved in migration and neurite outgrowth, was overexpressed with a fold change of 486%, whereas fibrillin-1 (FBN1) was decreased (Fig. 4B). 14–3-3 proteins gamma and zeta (YWHAG and YWHAZ) decreased (Fig. 4C). TUMF, involved in protein biosynthesis, was upregulated, whereas proteins assisting protein folding, as peptidyl-prolyl cis–trans isomerase FKBP10 (FKBP10) and thioredoxin domain-containing protein 5 (TXNDC5) were downregulated. Chaperones as 78 kDa glucose-regulated protein (HSPA5), endoplasmin (HSP90B1), LDLR chaperone MESD (MESD), and calreticulin (CALR) were less expressed in HPL-ASCs compared to SC-ASCs, whereas stress-70 protein (HSPA9) and heat shock protein beta-1 (HSPB1) increased (Fig. 4D). Ubiquitin C-Terminal Hydrolase L1 (UCHL1), a deubiquitinating enzyme (Fig. 4E), and metabolic proteins (ENO1, LDHA, PKM) (Fig. 4F) were down regulated in HPL-ASCs, whereas mitochondrial proteins as mitochondrial 10-formyltetrahydrofolate dehydrogenase (ALDH1L2) and dihydrolipoyllysine-residue succinyltransferase component of 2-oxoglutarate dehydrogenase complex (DLST) (Fig. 4G) were upregulated. Among transport proteins, leucine-rich repeat-containing protein 59 (LRRC59) increased, whereas cellular retinoic acid-binding protein 2 (CRABP2) were down regulated (Fig. 4H).

**Fig. 5:**
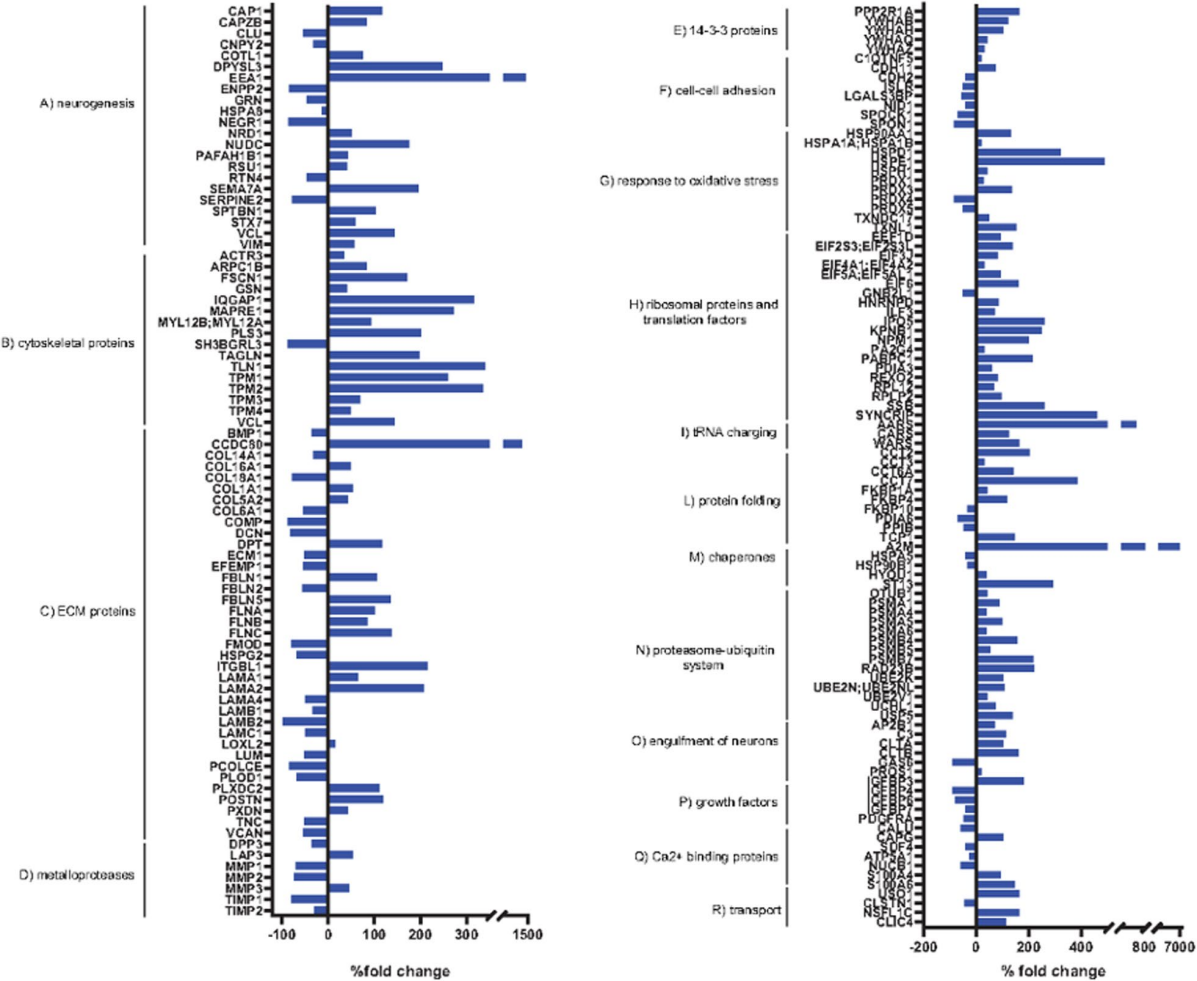
Proteomic analysis of conditioned media from HPL-ASCs vs SC-ASCs. Bars of differentially expressed proteins after label-free quantification (LFQ intensity % variation, *p*-value < 0.05, *q*-value < 0.05) were displayed

The secretome analysis highlighted the upregulation of proteins involved in axon development and guidance, as well as in neurite outgrowth and synaptogenesis in HPL-ASCs compared to SC-ASCs (Fig. 5A). Cytoskeletal proteins increased in HPL-ASCs secretome (Fig. 5B), indicating their relevance in driving neurogenesis, neurite branching and outgrowth. The great rearrangement of ECM proteins (Fig. 5C) suggested their primary role in modulating the maintenance, proliferation, self-renewal and differentiation of stem cells. Matrix metalloproteinase proteins remodeling decreased except for MMP3, involved in the degradation of fibronectin, laminin, collagens 3, 4, 9 and 10 and cartilage proteoglycans (Fig. 5D). 14-3-3 proteins, highly expressed in the brain during development, were upregulated (Fig. 5E). Cell–cell adhesion proteins decreased except for complement C1q tumor necrosis factor-related protein 5 (C1QTNF5) and cadherin-11 (CDH11) (Fig. 5F). Proteins involved in oxidative stress response increased (Fig. 5G). A feature of the neural stem cells transition from a quiescent to an activated state is provided by upregulation of protein biosynthesis, as evidenced in HPL-ASCs (Fig. 5H). 60 s ribosomal proteins, translation initiation factors and elongation factors were upregulated, as well as proteins involved in tRNA charging (Fig. 5I) and protein folding (Fig. 5L, M). In addition, proteins of proteasome-ubiquitin system, required for neural progenitor cell self-renewal, pluripotency and differentiation, increased in HPL-ASCs compared to SC-ASCs (Fig. 5N). Proteins involved in the internalization of synapses and neural progenitor cells by microglia, also increased (Fig. 5O). Among insulin growth factors, only IGFBP3 was upregulated (Fig. 5P). Ca2+ binding and transport proteins increased except for 45 kDa calcium-binding protein (SDF4), mitochondrial ATP synthase subunit alpha (ATP5A1), nucleobindin-1 (NUCB1) and calsyntenin-1 (CLSTN1) (Fig. 5Q, R).

### DRG neurite outgrowth is stimulated in the presence of HPL-ASCs more than SC-ASCs

**Fig. 6:**
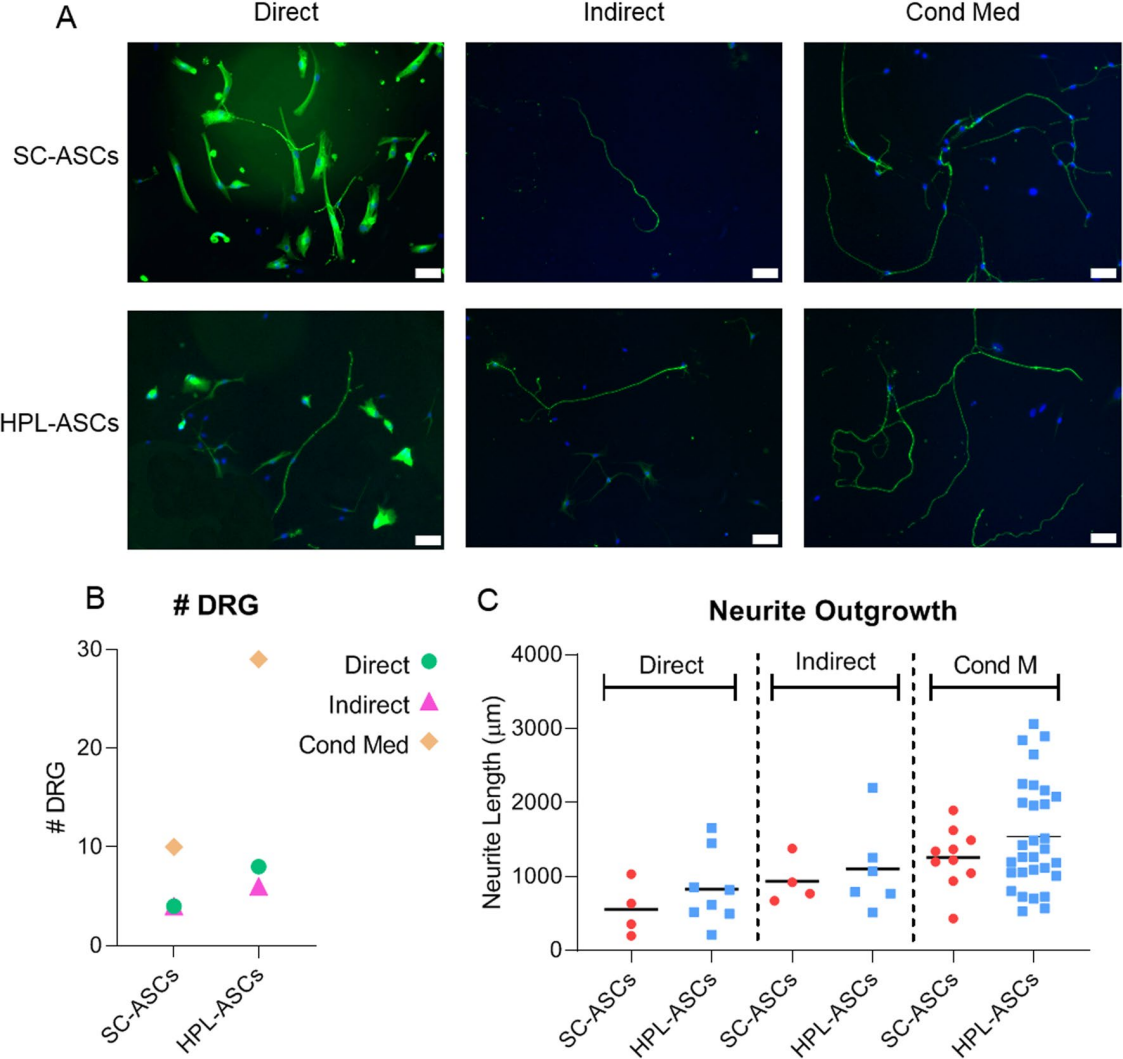
Functional co-culture of DRG with SC-ASCs or HPL-ASCs or their conditioned medium. **A** DRG were co-cultured in three different conditions: directly in contact with SC-ASCs/HPL-ASCs; indirectly with SC-ASCs/HPL-ASCs; alone with the conditioned medium obtained from SC-ASCs/HPL-ASCs. **B** the number of the DRG at the end of the co-culture indicated a higher survival rate in the presence of HPL-ASCs compared to the corresponding condition with SC-ASCs. **C** the total length of the neurites for each DRG revealed that the outgrowth was directly related to the specific condition and increased in the presence of HPL-ASCs. The neurites were the shortest in the direct co-cultures, they increased in the indirect co-cultures, reaching its maximum in the conditioned medium condition (white bars: 100 μm)

To understand if the effect exerted by HPL on neurotrophic properties of ASCs was not only phenotypical but also functional, we set up a co-culture with primary DRG and SC-ASCs or HPL-ASCs. In particular, three different culture conditions were evaluated: direct, indirect and in the presence of conditioned medium obtained from both SC-ASCs and HPL-ASCs (Fig. 6A). Interestingly, at the end of the culture period the number of DRG survived was higher when they were cultured in the presence of HPL-ASCs compared to the corresponding condition of SC-ASCs, especially in the presence of the conditioned medium (Fig. 6B). To differentiate the effect exerted by HPL-ASCs from HPL itself, DRG were cultured also alone in the presence of HPL medium. Despite the high amount of neuronal growth factors contained in HPL, DRG alone did not survive until the end of the culture, suggesting that the interaction HPL-ASCs was fundamental for promoting DRG survival. Moreover, the analysis of the DRG neurite outgrowth revealed that the length was directly related to the specific condition. In fact, the neurites were the shortest in the direct co-cultures, they increased in the indirect co-cultures, reaching its maximum in the conditioned medium condition (Fig. 6C). These data confirm that whether in presence of FBS or HPL the neuroregenerative properties of ASCs are mainly mediated by their secretome than the cells themselves, paving the way for an innovative stem cell therapy off-the-shelf. Furthermore, within each condition the neurite outgrowth was higher in the presence of HPL-ASCs or their conditioned medium compared to SC-ASCs (Fig. 6C), supporting our theory that HPL could stimulate ASCs neurotrophic properties and exert in turn their neural regenerative effect at a level even more than SC-ASCs.

## Discussion

PNI is a common issue that affects more than 1 million people every year worldwide and causes significant motor disabilities and chronic pain, with potentially devastating impacts on social and economic conditions [3, 41]. Currently, PNI with no nerve tissue loss or a short nerve gap is commonly treated through a surgical nerve coaptation, while for longer nerve gaps there is no consent yet on a recognized resolutive therapy [1]. Therefore, an innovative therapy respecting the human-translatable regulations is needed and, in this context, ASCs are emerging as a promising strategy to promote tissue regeneration [12–15]. According to the GMP guidelines, the use of FBS for ASCs culture should be abandoned in favor of xeno-free alternatives, such as HPL that presents less safety and ethical limitations [5, 29].

It has already been proven that ASCs showed a potential neurotrophic support when differentiated into SC-ASCs both in vitro and in vivo [16, 21, 42]. However, after the removal of the induction factors, SC-ASCs very rapidly underwent reversion of their phenotype, indicating a dedifferentiation process and suggesting a fragile differentiation method [21, 43, 44]. In addition, the differentiation protocol is time consuming with limited clinical translation potential and high-costly [12]. Considering also that the resulting benefits are not clear, the in vitro differentiation of ASCs is an uncompleted topic and SC-ASCs should be overcome in future therapies [18]. A promising alternative could be represented by culturing ASCs in the presence of HPL. Indeed, we have already demonstrated the important role of HPL in enhancing the neurotrophic properties of ASCs compared to undifferentiated FBS-cultured ASCs [14]. With this in mind, we wanted to analyze if neurotrophic properties of HPL-cultured ASCs could be even better than SC-ASCs differentiated by growth factors enhancement in the presence of FBS.

Among the different neurotrophic factors physiologically released by SCs after a nerve injury, NGF in particular has a robust neuroprotective effect [44]. Indeed, since its discovery by Rita Levi Montalcini in 1954 it was clear that NGF exerted a trophic effect essential in neural development, function and during peripheral nerve repair, stimulating neuronal survival and promoting axonal growth and elongation [45, 46]. Remarkably, we found out that the expression at transcriptional level of NGF was higher in HPL-ASCs compared to SC-ASCs, suggesting that HPL could enhance the ASCs neurotrophic properties and in turn, their neuroprotective action during nerve repair processes.

Together with the increasing of NGF, also nestin is essential for renewal, survival, and proliferation of neural progenitor cells. The enhancing of this protein in HPL-ASCs further supports the stimulation of neurotrophic properties compared to SC-ASCs. Moreover, nestin is a known neuronal precursor cell marker and it is expressed only during early stages of the development in the differentiating neural crest stem cells and its expression decreases with the progress of differentiation [47–49]. We supposed that HPL maintained ASCs at an early stage of neuroinduction compared to completely differentiated SC-ASCs. Proteomic analysis not only confirmed the increase in nestin but also of other cytoskeletal proteins, including alpha-actinin-4 (ACTN4), responsible for filopodia formation and highly expressed in neural stem/progenitor cells (NSPCs), and filamin A (FLNA), involved in neural progenitor migration and proliferation [50, 51].

The neuronal progenitors undergo a series of fine cytoskeletal-driven morphological changes during differentiation processes (i.e., neuritogenesis, axonogenesis and dendritogenesis) and the cytoskeletal reorganization itself is controlled by the interaction with the ECM, which in turn modulates proliferation, self-renewal and differentiation of stem cells, regulating the shape of axons and dendrites and neurite extension [52–55]. Among ECM proteins increased in HPL-ASCs, fibronectin is particularly interesting since it promotes neurite outgrowth and axonal regeneration, improving neurotrophic capabilities of ASCs cultured in the presence of HPL [5, 55–57]. Moreover, ALDH1L2 and ALDH have a role as ROS scavenger and are considered as markers for stem cells. The overexpression of ALDH1L2 in HPL-ASCs confirmed the hypothesis of an initial neuro-induction phase which keep unaltered the stem properties of ASCs [58, 59].

SCs have various developmental stages, each characterized by distinct specific markers [60]. During embryonic development, SCs originate from neural crest cells, which differentiate into SCs precursors, immature SCs and subsequently in pro-myelin SCs. Afterward, two types of matured SCs are formed, that is myelinating SCs and the non-myelinating SCs [59]. Among the different expressed proteins, S100B is widely used as specific SCs marker even though its expression is closely related to the maturation of SCs and may not be expressed in SCs precursors [61, 62]. Indeed, SCs precursors lack the expression of S100B and immunofluorescence staining data showed that in the early stage of culture, they do not totally express S100B [61]. In agreement with these results, we did not detect the expression of both mRNA and protein of S100B, supporting the hypothesis of an immature state of HPL-ASCs. Together with S100B, NGFR is considered a SCs specific marker, which was higher expressed in SC-ASCs, indicating the effective differentiation into SCs [63]. Conversely, immature SCs fail to express this marker and immunofluorescence data showed that in the early stage of culture SCs do not express NGFR and similarly to S100B its expression increases throughout the passages [61, 64]. Consistently with the expression of S100B, NGFR was less expressed in HPL-ASCs compared to SC-ASCs, further supporting the immature stage of ASCs induced by HPL without promoting their complete differentiation.

The secretome from stem cells is a critical component of how they regulate their proliferation and self-renewal [65]. Stemness is regulated by protein homeostasis (proteostasis) with a proper balance between protein biosynthesis and clearance [66, 67]. This is highlighted in the secretome from HPL-ASCs, that showed higher levels of ribosomal proteins, translation and elongation factors, as well as tRNA charging proteins. Components of the ATP-dependent chaperonin-containing T-complex (TRiC), TCP1, CCT2, CCT7, CCT3 and CCT6A, promoting protein folding or clearance to maintain proteostasis also increased, as for NSPCs [67]. α2-Macroglobulin (A2M) is a clearance factor involved in the degradation of extracellular misfolded proteins and promotes the regeneration of stem cells and also influences ECM remodeling by inhibiting the protease activity of MMP-2, actually decreased in HPL-ASCs [68–70]. Proteins involved in proteasome ubiquitin system were upregulated in HPL-ASCs, thus promoting self-renewal. Phagocytosis of dying neurons or exuberant neuronal branches by microglia resulted to be increased [71]. Proteins inhibiting oligodendroglial differentiation such as metalloendopeptidase BMP1 and tenascin C were down regulated in HPL-ASCs [72–74]. These data strongly support our theory that HPL maintained the stem properties of ASCs, while being in an initial neuro-induction phase that gives them more neurotrophic properties.

The development grade of SCs is evaluated also through their myelinating activity. MBP is a myelination-associated marker localized at the membrane level in active myelinating cells and with an expression rate increasing during the maturation of SCs [75, 76]. MBP is absent in SCs precursors and it is present at low levels in immature SCs and higher in mature and active myelinating SCs [61]. Consistently, we saw that MBP signal in HPL-ASCs was weak and distributed in the cytosol, while in the SC-ASCs the signal was dotted, compatible with active membrane proteins, indicating an early stage neuro-commitment of HPL-ASCs compared to completely differentiated SC-ASCs.

The increasing of the neuroregenerative properties of HPL-ASCs was evident also in an in vitro functional model in the presence of DRG. Indeed, we detected a higher number of DRG survived until the end of the assay and a longer total neurite length in the presence of HPL-ASCs compared to SC-ASCs, suggesting that culturing ASCs with HPL might be synergistic and more beneficial rather than differentiate them in SC-ASCs. Although HPL presents a high concentration of trophic molecules, it did not sustain nerve regeneration on its own, as demonstrated also by a previous published paper [5]. With the support also of our results, we can assert that HPL exerts an indirect effect on DRG enhancing ASCs neuroregenerative properties.

The neurotrophic effect of HPL-ASCs was particularly evident on DRG cultured with the conditioned medium, further indicating the importance of ASC-released neurotrophic factors induced by HPL, including NGF, which is considered fundamental in promoting axonal growth [77, 78]. Prautsch et al., demonstrated that DRG exhibited a significant and dense axonal outgrowth in response not only to direct NGF stimulation but also to the secretome resulting from NGF-stimulated ASCs, indicating the fundamental function of NGF and the all ASCs secretome as well [78]. Similarly, we found that HPL stimulated the expression of NGF and, in turn, the neurite outgrowth, further supporting the use of HPL-stimulated ASCs instead of differentiated SC-ASCs. Curiously, we are in contrast with a previous published paper evaluating the same functional assay where the authors observed an axonal outgrowth significantly higher in direct co-cultures compared to indirect co-cultures [5]. This discrepancy could be explained by the different systems used in the experimental set up. In fact, while the DRG used for the experiments described in this paper were purchased as single cells, the DRG used by Guiotto et al. were freshly isolated from explants and directly seeded with ASCs.

The great neurotrophic potential of HPL-ASCs secretome was confirmed by proteomic analysis, with the upregulation of proteins involved in axon development and guidance, as well as in neurite outgrowth and synaptogenesis. Among these proteins, we highligth early endosome antigen 1 (EEA1), which is highly expressed in the postsynaptic neurons and it is involved in synaptic plasticity, and semaphorin 7A (SEMA7A), promoting axon outgrowth through integrins and MAPKs and mediating the action of dihydropyrimidinase-related protein 3 (DPYSL3) [79–81]. Consistently, reticulon-4 (RTN4), known as a potent neurite growth inhibitor, was decreased [52]. Proteins involved in neuronal differentiation often regulate cytoskeletal organization (i.e., vinculin (VCL), vimentin (VIM), capping actin protein of muscle z-line subunit beta (CAPZB), and coactosin like f-actin binding protein 1 (COTL1) or ECM remodeling, confirming the relevance of these structural compartments as active players of cell fate determination, crucial in neurogenesis to ensure the development of the correct shape and function. Laminins are key components of ECM and have been associated with promoting neurite outgrowth [82]. Coiled-coil domain containing 80 (CCDC80), increased in HPL-ASCs, has been proposed to be a component of the ECM able to bind various ECM proteins and promoting cell adhesion [82, 83].

## Conclusions

Our data suggest that HPL is an optimal supplement to culture ASCs for PNI treatment, improving their neurotrophic properties and at the same time keeping them in a neuroinduced state while preserving their stem properties. This is particularly important when HPL-ASCs are considered as potential candidate for injective treatments in the contest of regenerative therapy. Considering that ASCs exert their regenerative effect mainly by the activation of resident stem cells through the secretion of several bioactive molecules, the undifferentiated state of HPL-ASCs makes them more competent in promoting the regeneration process at the injured site [84].

The efficacy of ASCs injection approaches may be impaired by cell manipulation, and its wide application is strongly limited by regulatory issues [84, 85]. Therefore, the use of HPL-ASCs could represent a real advantage since they need fewer manipulation steps compared to the differentiation of FBS-cultured ASCs into SCs, with the consequent advantages of reducing time and costs in term of expansion and differentiation protocols. Moreover, considering our results and previous reports indicating that the therapeutic ability of ASCs is mainly related to the secretion of biologically active factors the use of products based on conditioned medium obtained from HPL-ASCs represents a promising strategy for a “off-the-shelf” therapy in the contest of nerve repair [84].

## Data Availability

The datasets generated and analyzed during the current study are available in the OSF repository (https://osf.io/cd64e/?view_only=d1ddfe7f13764578af0fb80bff01a3f1).

## Abbreviations

ASCs: Adipose-derived stem cells
BDNF: Brain-derived neurotrophic factor
bFGF: Basic fibroblast growth factor
DRG: Dorsal root ganglia
HPL: Human platelet lysate
HPL-ASCs: HPL-cultured ASCs
NGF: Nerve growth factor
PDGF: Platelet-derived growth factor
PNI: Peripheral nerve injury
SC-ASCs: SC-like ASCs
SCs: Schwann cells
TGF-α: Transforming growth factor-α
VEGF: Vascular endothelial growth factor
ECM: Extracellular matrix
GMP: Good manufacturing practice
MBP: Myelin basic protein
NGFR: Nerve growth factor receptor
